# Community prevalence of chronic respiratory symptoms in rural Malawi: Implications for policy

**DOI:** 10.1371/journal.pone.0188437

**Published:** 2017-12-07

**Authors:** Hastings T. Banda, Rachael Thomson, Kevin Mortimer, George A. F. Bello, Grace B. Mbera, Rasmus Malmborg, Brian Faragher, S. Bertel Squire

**Affiliations:** 1 Research for Equity and Community Health Trust, Lilongwe, Malawi; 2 Collaboration for Applied Health Research & Delivery, Liverpool School of Tropical Medicine, Liverpool, United Kingdom; 3 LHL International, Oslo, Norway; University of Cape Town Lung Institute, SOUTH AFRICA

## Abstract

**Background:**

No community prevalence studies have been done on chronic respiratory symptoms of cough, wheezing and shortness of breath in adult rural populations in Malawi. Case detection rates of tuberculosis (TB) and chronic airways disease are low in resource-poor primary health care facilities.

**Objective:**

To understand the prevalence of chronic respiratory symptoms and recorded diagnoses of TB in rural Malawian adults in order to improve case detection and management of these diseases.

**Methods:**

A population proportional, cross-sectional study was conducted to determine the proportion of the population with chronic respiratory symptoms that had a diagnosis of tuberculosis or chronic airways disease in two rural communities in Malawi. Households were randomly selected using Google Earth Pro software. Smart phones loaded with Open Data Kit Essential software were used for data collection. Interviews were conducted with 15795 people aged 15 years and above to enquire about symptoms of chronic cough, wheeze and shortness of breath.

**Results:**

Overall 3554 (22.5%) participants reported at least one of these respiratory symptoms. Cough was reported by 2933, of whom 1623 (55.3%) reported cough only and 1310 (44.7%) combined with wheeze and/or shortness of breath. Only 4.6% (164/3554) of participants with chronic respiratory symptoms had one or more of the following diagnoses in their health passports (patient held medical records): TB, asthma, bronchitis and chronic obstructive pulmonary disease)

**Conclusions:**

The high prevalence of chronic respiratory symptoms coupled with limited recorded diagnoses in patient-held medical records in these rural communities suggests a high chronic respiratory disease burden and unmet health need.

## Introduction

Both non-communicable chronic respiratory diseases (including chronic obstructive pulmonary disease [COPD], asthma, bronchiectasis) and tuberculosis (TB), cause a major burden of disease and challenge to public health in developing countries and are on the increase globally [1–4]. The burden is compounded by under- or mis-diagnosis and/or under-reporting at primary health care level [5]. COPD prevalence estimates fall between 4.1% - 24.8%[6]. Asthma, a neglected, but increasing public health problem in sub-Saharan Africa affects 300 million people of all ages and ethnic backgrounds globally; causing 250,000 deaths annually [5].

Chronic respiratory symptoms are common in the general population but weak primary health care systems in resource-poor countries are often unable to diagnose COPD, asthma, bronchiectasis [2],[7]. Factors contributing to low rates of diagnosis include limited access to, negative perceptions of quality of, and lack of diagnostic capability in, health care facilities by those with respiratory symptoms in rural populations. Lack of and/or delayed access to health care exacerbates debilitation and poverty for individuals with chronic symptoms, thereby impacting on their quality of life [8].

Malawi has a well established TB control programme with a functioning network of smear microscopy and GeneXpert facilities for diagnosis and a public health focus at primary care level on TB diagnosis and delivery of curative TB services. However only 10–20% if patients presenting at primary health care centres with persistent cough have TB [9]. The remainder are likely to have non-communicable, chronic respiratory conditions but are often frustrated because of the lack of diagnosis or treatment options. There is only one fully trained respiratory physician in public service in Malawi. Neither spirometry nor inhaler therapy are available in district or primary care level facilities and staff are unfamiliar with chronic non-communicable respiratory disease. If made, diagnoses of chronic non-communicable respiratory diseases are syndromic; based on symptoms and signs. No models of health service delivery for life-long chronic respiratory conditions have yet been developed for implementation and scale-up.

The underlying non-communicable causes of chronic respiratory symptoms in rural settings of Malawi are poorly understood. Tobacco is widely grown as a cash crop however few adults smoke. In the 2015–2016 Demographic Health Survey [10] smoking of any sort of tobacco amongst rural residents aged 15–49 was reported by 0.7% of women and 12.1% of men. While smoking prevalence is clearly higher amongst men, most (73%) report smoking fewer than 10 cigarettes per day [10] Although few adults smoke, 98% of households use some sort of solid fuel for cooking, with virtually all being wood [10]. Some of this cooking takes place in the main household, but 60% takes place in a building separate from the main household and still exposes adults to woodsmoke. Overall, therefore, underlying causes of respiratory symptoms are likely to include Chronic Airways Disease (CAD–comprising COPD, asthma, bronchiectasis), but capacity to make these diagnoses is very limited.

We conducted a cross-sectional survey to determine the prevalence of chronic respiratory symptoms and the proportion of symptomatic individuals with related diagnoses (CAD or TB) documented in their health passports. This work was conducted in rural communities of Dowa and Ntchisi districts in Malawi ahead of a cluster randomised trial [11] registration number PACTR: PACTR201411000910192 in order to inform sample size calculations and implementation plans for the trial.

## Methods

### Study setting

A cross-sectional study was conducted between September 2014 and March 2015 in 27 rural primary health centres (total population of 640,000) in two rural districts; Dowa and Ntchisi (total population of 781,000) in Central Malawi. Malawi is a land-locked, low-income country located in the south-eastern region of Sub-Saharan Africa.

Currently in Malawi, there are three tertiary referral hospitals (Lilongwe, Blantyre and Zomba), twenty seven secondary level district hospitals (one per district) and 460 primary health care services (health centres, community hospitals and health posts) within each district [10]. Primary health centres are the first port of call for rural patients before they can be referred to district hospitals.

### Participants

Potential participants were approached in their homes after a population proportional sampling process (see below). Eligibility criteria were: resident in the household, aged 15 years and above, and able to give consent. We excluded individuals below the age of 15 years, those who refused to participate and visitors. Consent was obtained, first from the village head, then from the head of household and from the eligible household resident.

### Ethics

The study obtained ethical approval from Research Ethics Committees of College of Medicine, Malawi, reference number P.07/13/1424 dated November 28^th^ 2013 and Liverpool School of Tropical Medicine, United Kingdom reference number 14.013RS dated July 22 2014.

### Sample size and sampling procedures

The sample size for this survey was determined to estimate the proportion of individuals aged 15 years and above with (a) evidence of chronic cough/wheeze/shortness of breath, and (b) a diagnosis in their health passport to a pre-determined level of precision. For the study population, it was assumed that (a) the mean household size was 4.6 [10] and (b) the proportion of individuals aged 15 years or more was 52%, so the expected (average) number of individuals aged 15 years and over per household would be 4.6 × 0.52 = 2.4.

Health centres with their catchment populations constituted the study clusters. A population-proportional sampling procedure based on electronic satellite maps was used. Details are published elsewhere [12] and included three stages:

▪ Stage 1: a random sample of 27 clusters / health centres were selected and 9 of these were randomly allocated to each of the 3 study arms.
▪ Stage 2: 30 villages were randomly selected from each of the 27 clusters based on sampling probabilities proportional to village population—Google Earth Pro (Google Inc., Mountain View, CA, USA) was used to define villages and estimate their sizes–this provided a total sample of 810 villages (270 per study arm).
▪ Stage 3: a simple random sampling procedure was used to select 7 households from each selected village, giving a total of 5,670 households (1,890 households per arm).

Based on the estimated 2.4 eligible persons per household, this was expected to provide a total eligible sample of 5,670*2.4 = 13,608 individuals (4,536 per study arm).

▪ Assuming a population prevalence of chronic wheeze / cough / shortness of breath of 10%, this sample size was sufficient to estimate the true prevalence in each of the study arms individually with a precision of ±0.87%; ignoring clustering effects, the 13,608 individuals in all 3 study arms combined would provide a precision of ±0.50%; adding a design effect size adjustment of 2 to allow for clustering effects, the combined sample would provide a sample precision of ±0.71%.
▪ Assuming that 5% of individuals identified with symptoms of chronic cough / wheeze / shortness of breath would have a diagnosis of CAD or TB in their health passport, the anticipated sample would provide an estimate of the true prevalence in each individual study arm with a precision of ±2.00. Ignoring clustering effects, the 3 study arms combined would provide a precision of ±1.17%; adding a design effect size adjustment of 2 to allow for clustering effects, the combined sample would provide a precision of ±1.64%.

### Data collection

During data collection selected households were traced using Samsung Galaxy S3 smartphones installed with GPS Essentials. There were two sources of data. Firstly, interviews, conducted using a questionnaire S1 Questionnaire which was uploaded onto smart phones. This made the large number of questionnaires easily manageable, provided real-time data entry and afforded internal validity and consistency checks. Trained research assistants administered the questionnaires and training included emphasis on re-checking participants’ understanding of the symptoms of interest, re-inforcing the questions with local descriptions of symptoms for example ‘kupuma konveka kakwiu’ (literally ‘breathing with a sound like ‘kakwiu’) for wheezing. At the end of each day, data in smartphones were checked by supervisors. Once checked, data were then electronically transferred from the smartphones to a server at central office for storage [12].

Secondly, data on the diagnoses of COPD/bronchitis, asthma, bronchiectasis and TB, were sourced from the patient-held health passports of those with respiratory symptoms. Pages within health passports which showed respiratory symptoms, CAD/TB diagnoses and prescribed drugs were photographed using the smartphones. Photographed pages with respiratory symptoms, CAB/TB diagnoses and prescriptions were validated by clinicians.

### Data analysis

For security, and to prevent analytical biases, all participant identifiers were removed from the electronic survey database prior to transfer to Microsoft Excel 2013 for cleaning and creation of generated variables S1 Data. Photographic data were reviewed by an independent clinician before being merged with the survey data base S2 Data. The cleaned and validated data base was transferred to Stata v.14 for computation of descriptive statistics for the participants’ socio-demographic information, respiratory symptoms, (chronic cough, wheezing and shortness of breath), care-seeking patterns and diagnoses of COPD/bronchitis, asthma, bronchiectasis and TB. Means and frequencies (with corresponding percentages) were computed for categorical variables.

## Results

Between September 2014 and March 2015, the research team visited 6309 households in 810 randomly selected villages situated within the catchment areas of 27 Health Centres. On average 585 respondents from 30 villages were interviewed per cluster.

**Fig 1:**
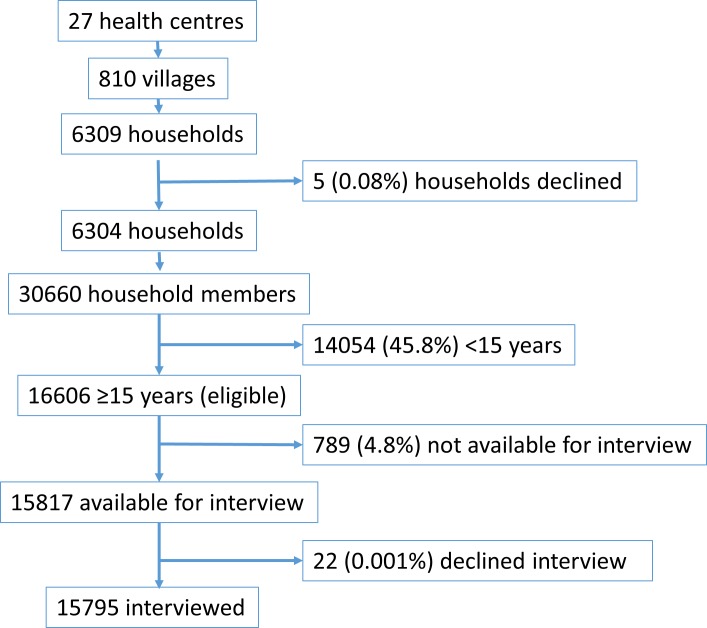
Flowchart illustrating process of selecting study participants.

Five households declined participation in the study. Remaining households were visited at least three times in order to maximise opportunities for interview. Out of the 30660 people in the remaining 6304 households, 14054 were <15 years old and therefore ineligible for interview. Of the remaining 16606 potential interviewees, 789 (4.8%) were unavailable during any household visit, having travelled away for either employment or business deals, and 22 declined interview citing lack of incentives and financial benefits. A total of 15795 people were interviewed, giving an individual response rate of 95.1% and household response rate of 99.9%. See Fig 1 for the whole participant selection process.

### Socio-demographic characteristics of respondents

Socio-demographic characteristics of respondents are shown in Table 1. There were 8150 (51.6%) female respondents and the mean (standard deviation) age was 35.6 (17.4) years. Participants’ ages ranged from 15 years to 99 years. Participants were predominantly from rural areas (99.5%) and dependent on subsistence farming for household income (87.9%). Many (68.3%) had attended primary school, but 16.6% had never attended school and only 14.7% and 0.4% had attended secondary school and tertiary education, respectively. Only 0.04% had access to electricity and 98.8% of participants used wood for cooking. Unfortunately the question about tobacco smoking was inadvertently omitted from the final uploaded version of the questionnaire.

**Table 1:** Socio-demographic characteristics of survey participants.

	N = 15,795	%
% female	8,150	51.6
Mean age of respondents	35.6 (SD = 17.4)	
Rural residence	**15,720**	99.5
Level of education		
None	2,628	16.6
Primary	10,779	68.3
Secondary	2,321	14.7
Diploma/degree	66	0.4
Main source of income		
Farming	**13,885**	87.9
Business	568	3.6
Casual work	468	3.0
Civil servant	263	1.7
Private sector	41	0.3
Craftsman	41	0.3
Remittances	193	1.2
Social cash transfer	1	0.0
Other	334	2.1
Main source of energy for cooking		
Paraffin/Kerosene	5	0.03
Charcoal	174	1.1
Firewood	**15,599**	98.8
Crop residues	9	0.1
Electricity	7	0.04

### Prevalence of chronic respiratory symptoms

**Fig 2:**
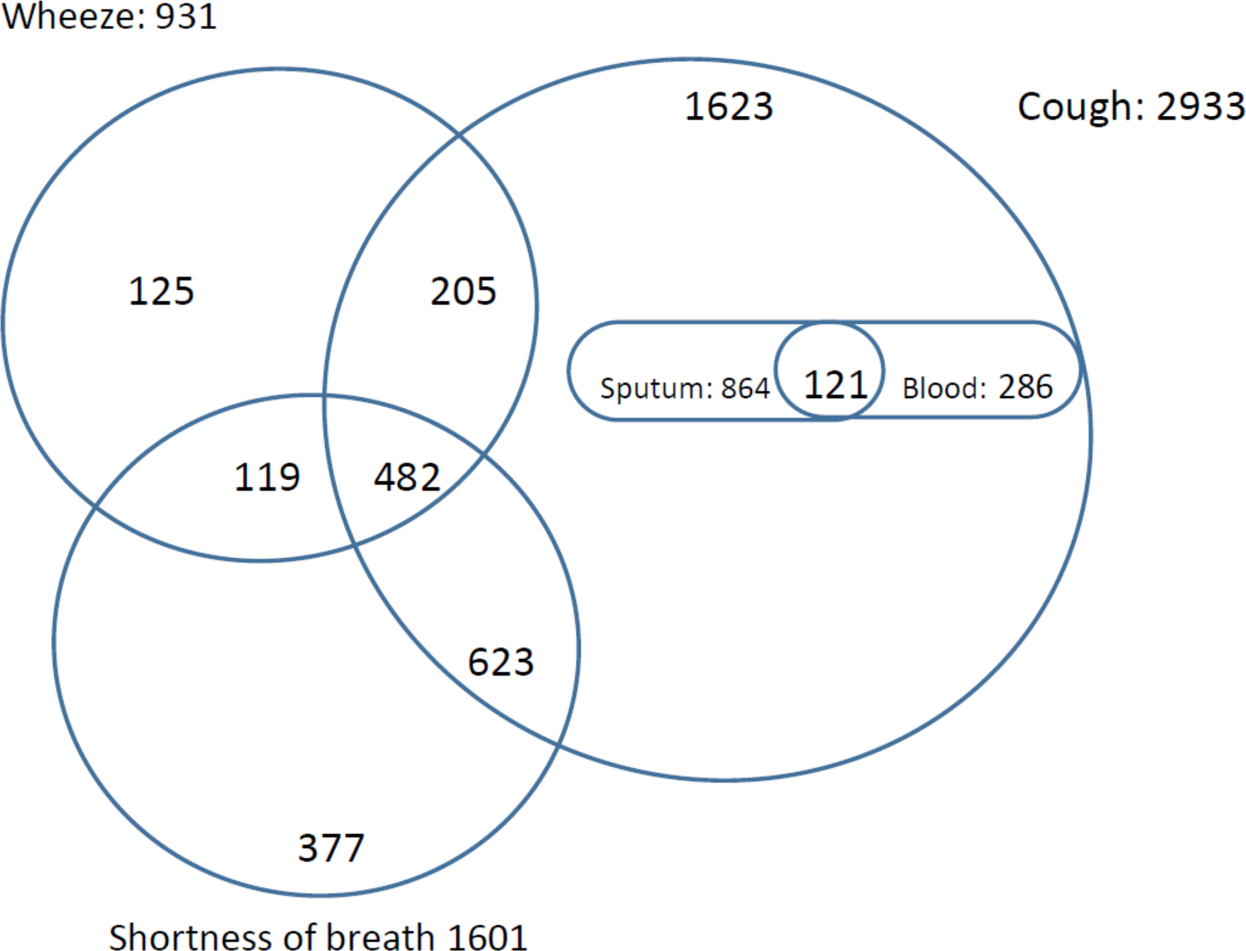
Prevalence and overlap of respiratory symptoms of chronic cough, wheeze and shortness of breath amongst 3554 respondents.

Out of the 15795 people interviewed, 3554 (22.5%) reported having experienced chronic cough, shortness of breath, or wheeze, either as individual symptoms or in some combination within the last 12 months. This is over twice the community prevalence of these symptoms that we predicted in our sample size calculation. Just over half of these symptomatic participants 1938/3554 (54.5%) were female. The inter-relationship between numbers of participants with one or more of these symptoms is illustrated in Fig 2.

There were 2933 participants (1609; 55% females) who experienced chronic cough, 1601 (904; 56% females) with shortness of breath and 931(460; 49% females) experienced wheezing. Among the 1623 who had chronic cough alone, 985 (61%) people reported cough productive of sputum, 121 (7%) reported blood in sputum and 286 (18%) reported coughing up blood without sputum. Interestingly there were 621 participants who did not experience chronic cough, reporting some combination of wheeze and shortness of breath.

### Diagnoses of chronic airways disease and tuberculosis in health passports

**Fig 3:**
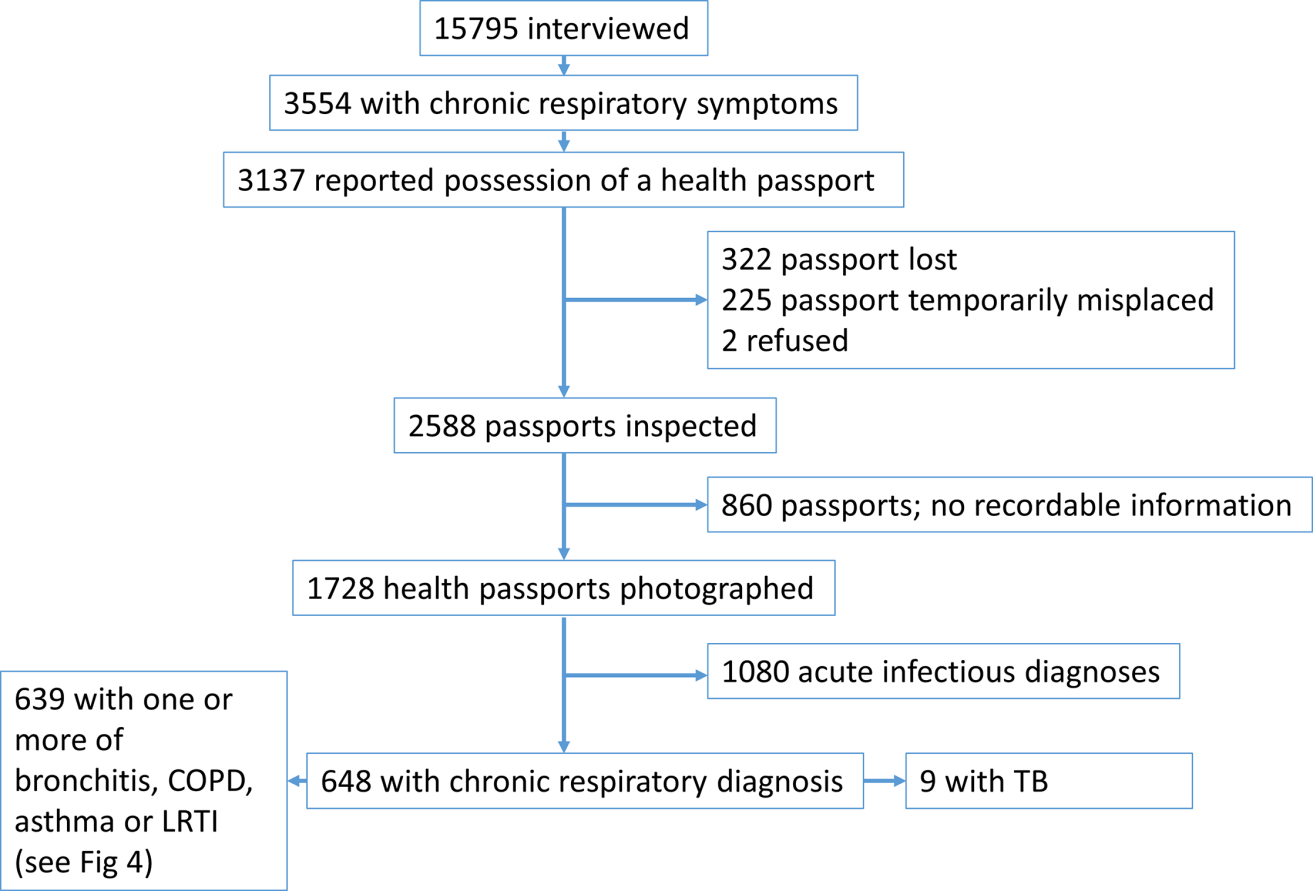
Health passport possession and recorded respiratory diagnoses.

The numbers of participants who reported possession of health passports, had recorded respiratory diagnoses, and the nature of those diagnoses is illustrated in Fig 3.

### Possession of medical records

Overall, 11018 (69.8%) respondents (6535 females 59.3%) with a mean age of 36.6 years (SD = 17.0) reported possessing a patient-held health passport. Of 3554 respondents who had experienced respiratory symptoms, 3137 (88.3%) reported possession of a health passport. Of these, 2 refused to have their passports checked, 322 had lost them completely and 225 could not locate them at the time of interview. Health passports from the remaining 2,588 participants were inspected. No relevant or recordable information was identified in 860 passports, leaving 1728 health passports which included at least one page from which respiratory symptoms and respiratory disease diagnoses could be captured.

### Recorded diagnoses for chronic respiratory symptoms

**Fig 4:**
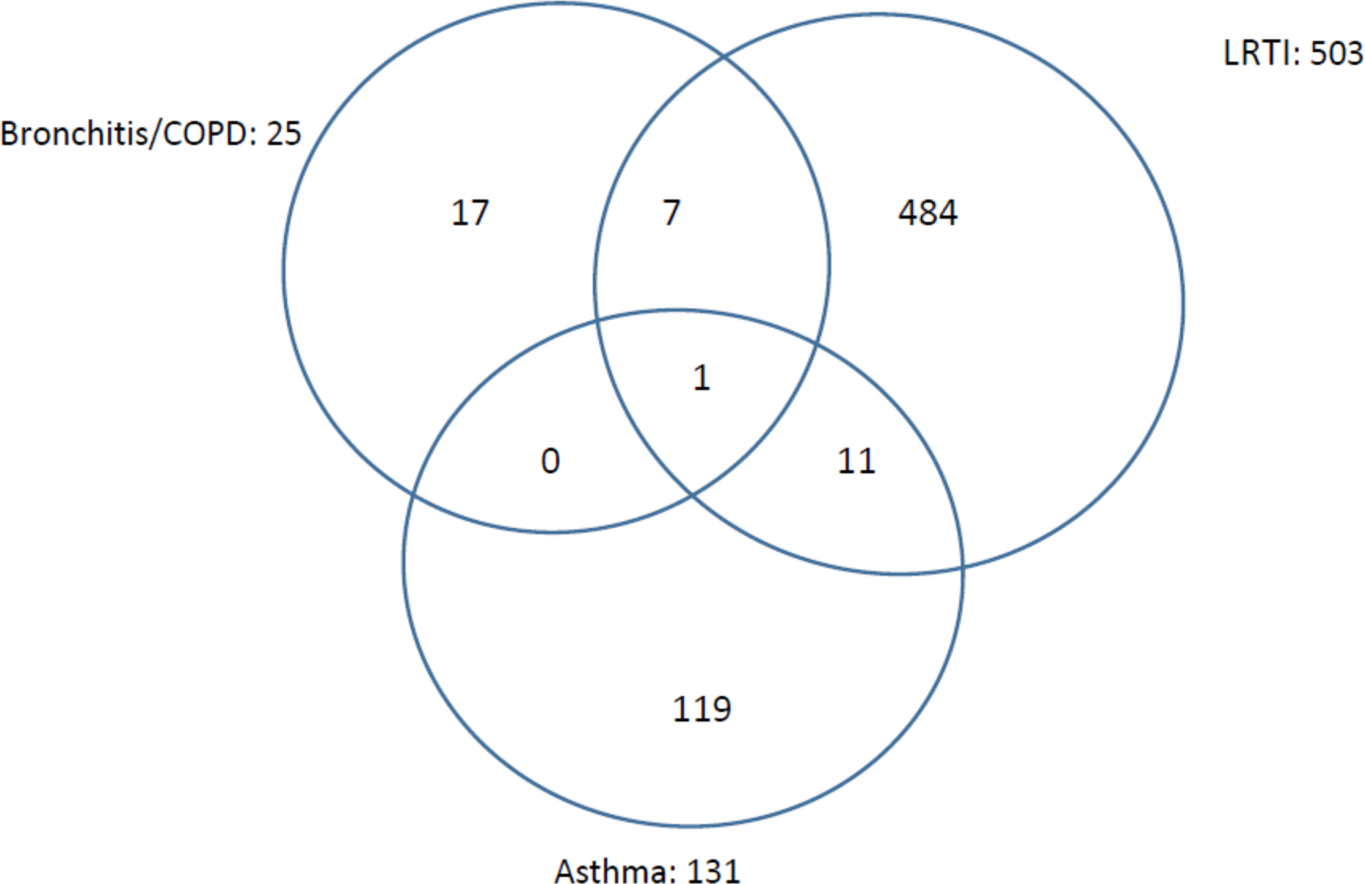
Chronic respiratory diagnoses recorded in health passports of 639 participants illustrating combinations and overlaps between diagnoses.

After exclusion of acute infectious diagnoses such as upper respiratory infections and pneumonia, 648/1728 (37.5%) participants had records of one or more of the following chronic, or potentially chronic diagnoses: lower respiratory tract infection (LRTI), TB, asthma, or bronchitis/COPD (See Fig 3). This group of diagnoses represents only 18.2% of the 3554 who had reported chronic respiratory symptoms. Overlapping diagnoses of COPD/bronchitis, asthma, and LRTI were seen but no overlap with tuberculosis was recorded. The different chronic respiratory diagnoses or combinations of diagnoses amongst the 639 participants with chronic respiratory diagnoses, but not TB, are illustrated in Fig 4.

Of 131 participants diagnosed with asthma (84 females), 119 had asthma alone, 11 overlapped with LRTI and one overlapped with COPD/bronchitis. Of 25 participants diagnosed with COPD/bronchitis (18 females), 17 had COPD/bronchitis alone, 7 overlapped with LRTI and one overlapped with asthma. LRTI was the most common diagnosis, being diagnosed in 503 participants (328 females). In 19 participants, LRTI was diagnosed in combination with asthma and bronchitis/COPD, but in 484 participants, LRTI was the sole diagnosis.

If the 484 participants who had LRTI alone are presumed not to have a documented CAD diagnosis, the number of patients with a diagnosis of either CAD or TB in their health passports falls from 648 to 164. Furthermore the proportion of patients with chronic respiratory symptoms who had a diagnosis of CAD or TB documented in their health passport falls from 18.2% (648/3554) to 4.6% (164/3554) which is close the estimate of 5% made in our sample size projection.

### Health care-seeking patterns

Participants with symptoms of chronic cough, wheeze and shortness of breath reported seeking care from various care providers: public or private health care units, as well as traditional healers and through self-treatment. Of 3554 symptomatic respondents 87.6% had sought care. Number and type of care-seeking visit are outlined in Table 2. Most visits were made to public health facilities, particularly the district hospital (2072 visits; 39%) or to pharmacy or grocery stores (1428 visits; 26.8%). Only a minority of visits were made to traditional herbalists (350; 6.6%).

**Table 2:** Number and type of care-seeking visit made by 3554 participants with chronic respiratory symptoms.

	No. of visits / occasions	% of visits / occasions
**Public health facilities**		
Central hospital	23	0.4
District hospital	319	6.0
Health centre	**2072**	39.0
Dispensary	159	3.0
**Private health facilities**		
Mission hospital	85	1.6
Private hospital/clinic	219	4.1
**Traditional**		
Herbalist	350	6.6
**Self**		
Pharmacy/grocery	**1429**	26.8
Self-treatment	540	10.1
**Religious healing**		
Prayers	128	2.4
**Totals**	**5324**^**+**^	100%

^**+**^Some participants made multiple visits to multiple facilities and care providers.

## Discussion

Our study is the first to clearly document the prevalence of chronic respiratory symptoms amongst adolescents and adults in rural Malawi. The prevalence, in the preceding 12 months, of 22.5% (3554/15795) of any of the three chronic respiratory symptoms: chronic cough, shortness of breath or wheeze is substantial and only 648/3554 (18.2%) of these symptomatic individuals had a diagnosis in their health passports.

### Chronic respiratory symptoms

Our findings are in line with previous population-based prevalence studies of respiratory symptoms which have been carried out in urban or peri-urban areas in Malawi. Piddock [13] found a high rate of charcoal use (81.5%) in peri-urban households as a predisposing factor to chronic respiratory symptoms. Our study shows 98.8% use of wood in rural households. Meghji [14] found more than 40% of Malawian adults in urban areas to have abnormal lung function. Unfortunately we omitted questions about tobacco smoking in the questionnaire. Although smoking prevalence is low in rural Malawi [10] this omission should be rectified in future work.

Our findings are also in line with data from health facilities. Chronic cough is the commonest presenting respiratory symptom both in Malawi [15] and in a number of other developing countries [16]. Data from nine countries surveyed by WHO in 2004, further show that 8.5% to 37% of patients aged above 5 years reporting to primary health care facilities sought care prompted by respiratory symptoms [17].

Cough, sometimes in association with other symptoms, was the commonest symptom in our study. Chronic or persistent cough has many causes [18] and is an important indicator of respiratory diseases including tuberculosis. Previous studies have found that a proportion of patients previously treated for TB will develop chronic respiratory disease [19].

Shortness of breath was the second most common symptom after cough. Previous work showed a prevalence of this symptom of 1 in 6 globally, in Sub-Saharan Africa the prevalence was 1 in 4 (25%) [15]. Shortness of breath is among the most common symptoms experienced in chronic obstructive pulmonary disease, worsening as the condition progresses and causing distress and disability [20]. High community prevalence of this symptom may indicate high prevalence of underlying lung or cardiac disease.

Wheezing was the third most common symptom. Wheeze is generally understood in relation to asthma, although the implications of the disease itself are not understood in rural communities [16]. In this study we found that wheezing overlapped with shortness of breath and chronic cough separately, and with both. The three symptoms occurring together might be confused with symptoms of other airway or cardiac diseases.

### Diagnoses in patient-held health passports

Chronic respiratory symptoms are associated with debility and lower health-related quality of life especially where no diagnoses are made and no clinical management offered as a result [21]. In turn this is likely to lead to lower productivity and poverty at household level. Only 648/3554 (18.2%) of symptomatic individuals in our study had a diagnosis in their health passport. LRTI was the commonest diagnosis recorded (503/648; 77.6%) and, judging by the overlap with diagnoses of COPD/bronchitis and asthma, may include many individuals with chronic airways disease who have been given the LRTI diagnosis for lack of familiarity among health care workers in this setting with any other diagnostic category. No participant was included in a long-term clinical management plan.

These findings reflect the substantial challenges faced by primary health care systems in developing countries with respect to diagnosis and management of asthma, COPD and other chronic respiratory diseases. Such challenges result from impeded access to primary health care and lack of staff and diagnostic equipment [22],[23], [24] among other factors. Participants in this study made multiple visits to public health facilities for free healthcare. However, the overall yield of people with a diagnosis was very low, and the majority received non-specific LRTIs diagnosis. Chronic respiratory diseases have been shown to be on the increase in developing countries and globally [25], and are an issue in urban Malawi [14]. However, in many developing countries, chronic respiratory diseases are not seen as a key health priority and therefore can be overlooked by governments and health professionals. Weak health systems and few diagnostic or treatment options mean patients and their families struggle to manage their disease.

### Implications for health system strengthening

The combination of finding a high prevalence of chronic respiratory symptoms and a low prevalence of recorded diagnoses argues strongly for the piloting of health service delivery improvements at primary care level.

## Supporting information

S1 Questionnaire Study questionnaire. This is the text version of the study questionnaire that was uploaded onto smartphones.
(DOCX)

S1 Data Dataset of reported symptoms. This is the Excel spreadsheet that contains the minimal dataset collected in responses to the study questionnaire.
(XLSX)

S2 Data Health passport data. This is the Excel spreadsheet that contains the diagnostic data that were extracted from the health passports.
(XLS)
