# Supplementary material for: Community prevalence of chronic respiratory symptoms in rural Malawi: Implications for policy
Source: PLoS One. 2017 Dec 7;12(12):e0188437. doi: 10.1371/journal.pone.0188437 (PMC5720679; doi:10.1371/journal.pone.0188437)
Supplement: S1 Questionnaire — This is the text version of the study questionnaire that was uploaded onto smartphones. (DOCX) [file pone.0188437.s001.docx]

| **Baseline Household Survey Questionnaire** | | | | | | | | | | | | |  |
| --- | --- | --- | --- | --- | --- | --- | --- | --- | --- | --- | --- | --- | --- |
| Date of interview:\|___\| ___\|___\|___\|__\|__ \| | | | | | | Interviewer’s Name: | | |  | | | |  |
|  |  |  |  |  |  | Respondent Number: | | |  | | | |  |
| **Question** | | | | | | **Options** | | | **Code** | | | |  |
| 1. ***Socio-demographic information*** | | | | | | | | | | | | |  |
| 1. Respondent’s relationship to  household head.  **(Kodi chibale chanu ndi mwini nyumba**  **ndi chotani?)** | | | | a. Head of household  b. Husband  c. Wife  d. Mother/Father  e. Son/Daughter  f. Other relative  g. Non relative | | | | | |  | | |  |
| 2. Respondent’s Sex.  **(Kodi oyakha mafunsoyo ndi wam’munaKapena wamkazi?)** | | | | a. Male  b. Female | | | | | |  | | |  |
| 3. Respondent’s age is *(in complete years):*  **(Muli ndi zaka zingati?)** | | | |  | | | | | |  | | |  |
| 4. Respondent’s dwelling unit is in what  area?**( Amakhala dela lanji?)** | | | | a. Rural  b. Urban (Boma or Trading Center) | | | | | |  | | |  |
| 5. What is the respondent’s marital status?  **(Kodi muli pa banja?)** | | | | a. Married  b. Divorced /separated  c. Widowed  d. Never married | | | | | |  | | |  |
| 6. What is the highest qualification  attained by the respondent?  **(Kodi maphunziro anu munalekezera pati?)** | | | | a. None  b. Primary Level  c. Secondary Level  d. Diploma/Degree  e Postgraduate | | | | | |  | | |  |
| 7. What is your main source of income for  the household?  **(Kodi pakhomo panopa kwenikweni mumadalira chiyani kuti mupeze ndalama?)** | | | | a. Farming  b Business  c. Casual work  d. Civil Servant  e. Private Sector  f. Craftsman  g. Remittances  h. Social Cash Transfer  i. Other (specify) _________________________ | | | | | |  | | |  |
| 8. What is your occupation?**(Mumagwira ntchito yanji?)** | | | | a. Farming  b Business  c. Casual work  d. Employed  e. Student  f. Other (specify) _________________________ | | | | | |  | | |  |
| 9 a) How many people in total usually  sleepin this house?**(Nyumba inoyi mumagona anthu angati onse pamodzi?)**  b) State the number for each   age group**.(Ndi anthu angati azaka izi amene amagona mnyumba mwanumu?)** | | | | Total:___________________  <15 years old: | | | | | |  | | |  |
|  |  |  |  | 15 - 39 years old: | | | | | |  | | |  |
|  |  |  |  | 40+ years: | | | | | |  | | |  |
| 10. What is the main source of energy for  cooking in this household?  **(Kodi mumagwilitsa tchito njira yanji pophika nthawi zambiri?)** | | | | a. Paraffin/Kerosine  b. Charcoal  c. Firewood  d. Crop residues  e. Electricity  f. Dung  g. Liquid Petroleum Gas  h. Other (specify)_________________________ | | | | | |  | | |  |
| 1. ***DISABILITY SCREEN*** | | | | | | | | | | | | |  |
| 1. **Does the respondent have difficulty or problem in the following?** | | | | | | | | | | | | |  |
| 1. Seeing even if wearing glasses?   **(kodi mumavutika kuwona ngakhale mutavala magalasi amaso?)** | | | 1. No - no difficulty 2. Yes - some difficulty 3. Yes - a lot of difficulty 4. Cannot see at all | | | | | | |  | | |  |
| 1. Hearing even if using a hearing aid?**(Kodi mumavutika kumva ngakhale mutavala zothandizira kumva?)** | | | 1. No - no difficulty 2. Yes - some difficulty 3. Yes - a lot of difficulty 4. Cannot hear at all | | | | | | |  | | |  |
| 1. Walking or climbing uphill?   **(kodi mumavutika Kuyenda kapena kukwera chitunda?)** | | | 1. No - no difficulty 2. Yes - some difficulty 3. Yes - a lot of difficulty 4. Cannot walk or climb at all | | | | | | |  | | |  |
| 1. Remembering or concentrating?   **( kodi mumavutika kukumbukira kapena kukhala ndi chidwi pa zithu?)** | | | 1. No - no difficulty 2. Yes - some difficulty 3. Yes - a lot of difficulty 4. Cannot remember or concentrate at all | | | | | | |  | | |  |
| 1. With (self-care such as) washing all over or dressing?   **(Kodi mumavutika kudzisamalira monga kuzisambitsa kapena kuvala nokha?)** | | | 1. No - no difficulty 2. Yes - some difficulty 3. Yes - a lot of difficulty 4. Cannot care for myself at all | | | | | | |  | | |  |
| 1. Using the usual (customary) language, do you have difficulty communicating/speaking (for example understanding or being understood by others)?**(Kodi mumavutika kuyakhula, kumva kapena kuti anthu ena akumveni bwinobwino?)** | | | 1. No - no difficulty 2. Yes - some difficulty 3. Yes - a lot of difficulty 4. Cannot communicate at all | | | | | | |  | | |  |
| **If III or IV to any of the questions above, go to Question 2 and answer first then loop back to continue Question 1, otherwise go to CAD/TB Screen** | | | | | | | | | | | | |  |
| 2. a)What are the likely causes of this  condition?**(Kodi chinayambitsa vutoli ndi chiyani?)** | | | 1. Born with 2. Illness 3. Accident   4. Do not know | | | | | | |  | | |  |
| b) What is the duration (in years) of the  condition?**(Kodi vuto limeneli mwakhala nalo kwa nthawi yayitali bwanji? (in years)** | | |  | | | | | | |  | | |  |
| c) What is the status of the condition?  **(Nanga panopa mukupeza/kuona bwanji? (Pali kusintha kwina kulikonse?)** | | | 1. Getting better 2. No change 3. Getting worse | | | | | | |  | | |  |
| 1. ***CAD/TB SCREEN: Medical history (need specificity)*** | | | | | | | | | | | | |  |
| 1. Which of these symptoms have you had over the past 1 year?   **(Kodi ndi ziti mwa zizindikiro izi zimene mwakhala nazo kwa chaka chimodzi chapitachi?)** | | | a. Chronic/persistent cough other than when you have had a cold Yes □ No □ | | | | | | | |  | |  |
|  |  |  | b. Coughing Blood Yes □ No □ | | | | | | | |  | |  |
|  |  |  | c. Wheezing or whistling in the chest  Yes □ No □ | | | | | | | |  | |  |
|  |  |  | d. Shortness of breath when hurrying on the level or  walking up a slight hill  Yes □ No □ | | | | | | | |  | |  |
|  |  |  | e. Bringing sputum up from your chest other than  when you have had a cold  Yes □ No □ | | | | | | | |  | |  |
|  |  |  | f. Other(Specify)___________________________ | | | | | | | |  | |  |
| 1. ***PATIENT CONSENT SECTION***   The questions on this form are about patient's costs and are part of the assessment of patient treatment costs and coping strategies. | | | | | | | | | | | | |  |
| 1. Did you seek treatment or advice for these symptoms (in C1) at any of the following?   **(Kodi munapitako kukalandira chithandizo chilichonse chifukwa cha zizindikiro zomwe munatchula zija kumalo awa?)** | | 1. Central Hospital Yes □ No □ 2. District Hospital Yes □ No □ 3. Dispensary Yes □ No □ 4. Health Centre Yes □ No □ 5. Mission Hospital Yes □ No□ 6. Pharmacy, drug &Grocery store Yes □ No □ 7. Herbalist Yes □ No □ 8. Private hospital/clinic Yes □ No □ 9. Self-Treatment Yes □ No □   j. Prayers Yes □ No □ | | | | | | | | | | | |
| 1. Have you ever been treated for any of the following?   **(Kodi munalandilako chithandizo chamatenda awa?)** | | \| a) TB Yes □ No □ \| \| --- \| \| b) Asthma Yes □ No □ \| \| c) COPD Yes □ No □ \| \| d) Bronchiectasis Yes □ No □ \| \| e) Lower Respiratory Tract Infection Yes □ No □ (**If all NO, go to Question K1, If only one Yes, go to E1,**  **if multiple diagnoses then answer D6)** \| | | | | | | | | | | | |
| 1. May I see all your health passports or any medical records?   **(Kodi ndingawoneko kabuku kachipatala/kazaumoyo ngati muli nako?)** | | a) Yes  b) No, I don’t have  c) No, I don’t want  d) No, I lost it  e) No, I can’t retrieve it | | | | | | | | | | | |
|  |  |  |  |  |  |  |  |  |  |  |  |  |  |
|  |  |  |  |  |  |  |  |  |  |  |  |  |  |
|  |  |  |  |  |  |  |  |  |  |  |  |  |  |
|  |  |  |  |  |  |  |  |  |  |  |  |  |  |
| 1. a. Are the following diagnoses written in health passport or medical records?**(Kodi matenda awa alembedwa mukabuku ka zaumoyoka?)**   **(0 = no, 1 = yes for each)**  b. If there are no records of  diagnosis shown in the health  passport/medical records ,  explain why?(**Nchifukwa chani**  **simunalembedwe kalikonse**  **ka za matendawa mu kabuku**  **kachipatala/ ka zaumoyo?)** | | 1. TB | | | | | | | | | | | |
|  |  | 1. Asthma | | | | | | | | | | | |
|  |  | 1. COPD | | | | | | | | | | | |
|  |  | 1. Bronchiectasis | | | | | | | | | | | |
|  |  | 1. Lower Respiratory Tract Infection   Explain______________________________________________________ | | | | | | | | | | | |
| 1. What drugs are prescribed in health passport for the diagnoses made?**(Ndimankhwala anji omwe analembedwa mukabukuko kutengela ndi matenda omwe anapezeka nawo?)**   **(0 = no, 1 = yes for each)** | | 1. TB drugs | | | | | | | | | | | |
|  |  | 1. Salbutamol Inhalers | | | | | | | | | | | |
|  |  | 1. Inhaled Corticosteroid | | | | | | | | | | | |
|  |  | 1. Salbutamol tablets | | | | | | | | | | | |
|  |  | 1. Aminophylline tablets | | | | | | | | | | | |
|  |  | 1. Antibiotics | | | | | | | | | | | |
|  |  | 1. Other (explain) ______________   **If more than one diagnosis made, go to D6, if just one diagnosis made then go to Question E1.** | | | | | | | | | | | |
| 1. If more than one diagnosis was made, which was the most recent one?**( Ngati munalembedwa matenda oposela amodzi, ndi matenda ati omwe munthuyo anadwala chaposachedwa?)**    1. TB    2. Asthma    3. COPD    4. Bronchiectasis    5. Lower Respiratory Tract Infection | | | | | | | | | | | | |  |
| ***E. PREDIAGNOSTIC AND DIAGNOSTICS COSTS*** | | | | | | | | | | | | |  |
| 1. In Malawi Kwacha, about how much did you spend on the following before your most recent illness?   **(Kodi ndi ndalama zingati zomwe mwagwiritsa tchito pa izi achipatala asanakupezeni ndi ____ (*mention the most recent disease*?)** | - 1. Administrative costs:   2. Tests costs:   3. X-ray costs:   4. Drug costs:   5. Travel costs:   6. Food costs:   7. Accommodation cost: | | | | | | | | | | |  |  |
| **Coping Costs**  2. a) Did you borrow any money to cover costs  due to for your most recent illness?**(Kodi munatengako ngongole kuti mulipire mabill pamatenda amenewawa?)** | | | | | | | Yes □ No □**If No, go to question J3** | | | | | |  |
| **b)** If Yes, how much did you borrow?**(Munakongola ndalama zingati?)**___________________  **c)** From whom did you borrow? **(Munakongola kwa ndani?)**  **i)** Family: ……………… Yes □ No □ **ii)** Neighbours/friends:……Yes □ No □  **iii)** Bank: …… Yes □ No □ **iv)** Cooperative:………………Yes □ No □  **v)** Other: ………………Yes □ No □  **d)** If Other, specify:_______________________________________________________________  **e)**What is the duration of the loan?**(Kodi ngongoleyi inali yayitali bwanji?)** …………Weeks …………Months ………… Years (single answer)  **f)** Please indicate the intervals at which repayments are to be made:**(fotokozani kapelekedwe kangongoleyi)**  1) Weekly 2) Monthly 3) Annually 4)I am not expected to pay the money back  5) Other (specify):______________________________________________________________  **g)** What is the interest rate on the loan? (%) **(Nanga ngongoleyi inali ndi chiwongola dzanja chochuluka bwanji?)**  1) Less than 10 2) 10 to 15 3)More than 15 4) I don’t pay interest: | | | | | | | | | | | | |  |
| **3. a)** Have you sold any of your property to finance the cost of for your most recent illness?**(Kodi mwagulitsako katundu wina aliyense kuti mupeze thandizo chifukwa cha matendawa?)** | | | | | | | | Yes □ No □ | | | | |  |
| **b)** If Yes, what did you sell? **(Munagulitsa chain?)**  **i)** Land: ………………..Yes  No **ii)** Livestock: …Yes  No   **iii)** Vehicle:Yes  No **iv)** Household item:…….Yes  No   **v)** Farm Produce: ……Yes  No **vi)** Other: …………………Yes  No   **c)** If Other, specify:………………………………………………………………………………………………………………  **d)** How much did you earn from the sale of your property?:**(Kodi munapeza ndalama zingati mutagulitsa katunduyu?)**  **e)** What is the estimated market value of the property you sold?:**(Kodi katundu ameneyu pansika mukanamugulitsa ndalama zingati?)**…………………………………………………………………………………………… | | | | | | | | | | | | |  |
| ***F. TREATMENT COSTS SECTION*** | | | | | | | | | | | | |  |
| 1. Where do you currently get your drugs for your most recent illness?**(Kodi mankhwala anu mumakatenga kuti?)**   **(If the patient has visited two different health facilities places, tick the current place and report costs only for that place.**) | | | | | □Public Health Facility/hospital ……□Workplace □Community □Dispensary  □Private Health Facility/hospital…… | | | | | | | |  |
| 1. How many times a month do you go there to take your drugs?**(Pa mwenzi mumapita kangati kukatenga mankhwala?)** (select one answer)   □1 □2 □3 □4 □5 □6 □7 | | | | | | | | | | | | |  |
| 1. Who supports you when getting your drugs? **(Amakuperekezani ndi ndani pokatenga mankhwala?)**   (**select one answer**) | | | | | □Family member □HSA  □IHP □Self/no one | | | | | | | |  |
| 1. a) How do you get there? (indicate main means of transport)**(Mumayenda bwanji pokatenga mankhwala kumeneku?)** | | | | | - 1. Walking   2. Bicycle   3. Bicycle taxi   4. Ox-Cart   5. Motorcycle   6. Bus   7. Car   8. Other (Specify | | | | | | | |  |
| b) How long does it take to get there (in minutes)?**(Mumatenga nthawi yayitala bwanji kuti mukafike kotenga mankhwala?) (Mphindi)**  : | | | | | | | | | | | | |  |
| 1. How long does one of these visits take on average, including time on the road and waiting time?   (total turnaround time)_________________ minutes  **Kodi ulendo umodzi mongoyelekeza, nthawi yoyenda komanso yodikilira chithandinzo umakhala nthawi yochuluka bwanji? (ulendo onse kupita ndikubwela)**  _________________ minutes **(Mphindi)** | | | | | | | | | | | | |  |
| 1. From your home to the health facility, how much does it cost if you take transport (both ways)? **Kodi mumagwiritsa ntchito ndalama zingati ngati mwagwilitsa tchito transipoti kuchoka kunyumba kwanu kupita kuchipatala?)**   ________________________________ | | | | | | | | | | | | |  |
| 1. If you need to buy food (e.g. lunch), how much do you spend on food while travelling or waiting?   **(kodi mumagwiritsa ntchito ndalama zingati kugulira chakudya monga khomaliro mukamayenda kapena kudikilira chithandizo?** | | | | | | | | | | | | |  |
| 1. a) Do you have to pay administration fees when you go to receive treatment for your most recent illness?   **(Kodi munapereka ndalama monga zogulira kabukhu kakuchipatala, komanso kuti mukumane ndi adokotala m’mene munapita kukalandira chithandizo pamatenda omwe munadwala chaposachedwapa?)**    b) Do you have any accommodation costs when picking up your drugs for your most recent illness?**(Kodi munalipira malo ogona m’mene mukatenga mankhwala amatenda omwe mwadwala posachedwapa?)** | | | | | Yes □ No □  If yes, how much? ________________________  Yes □ No □  If yes, how much? ________________________ (Repeated Question) | | | | | | | |  |
| 1. a) Since the beginning of treatment , have   you ever had to go to the health facility in addition to your scheduled visits for follow up tests? ? **(Chiyambireni kulandira chithandizo, kodi munapitako kuchipatala kuti mukapimidwe kupatula maulendo omwe mumapita kukalandira chithandizo?)**  b) How long does one of these assessment visits take on average, including time on the road, waiting time and tests (total turnaround time)?…………………minutes  **(Kodi ulendo okapimidwawu mwachidule, umatenga nthawi yayitali bwanji kuphatikiza kuyenda, kudikila kuti mulandire chithandizo komanso kuti mupimidwe (Ulendo onse ndikubwelera kunyumb kwanu) in minutes)** | | | | | Yes □ No □  **If No, go to question G1** | | | | | | | |  |
| ***G. GUARDIAN COSTS SECTION*** | | | | | | | | | | | | |  |
| 1. Does any family/friend accompany you on any visits or go in your place to collect drugs for your most recent illness?   ?**(Kodi wachibale kapena nzanu anakuperekezanipo pamaulendo okatenga mankhwala m’mene munadwala?)** | | | | | Yes □ No □**If No, go to question H1** | | | | | | | |  |
| 1. On how many visits has your family/friend accompanied you or gone in your place   **(Ndi maulendo angati amene munthu ameneyu anakupelekezanipo kapena kupita m’malo mwanu?)**  **a)** For scheduled visits for assessmentfor your most recent illness/follow up?…□times  **(Maulendo ochita kukuuzani achipatala kuti mukayesedwe m’mene mukumvera m’mene munadwala pochedwapa)**  **b)** For unscheduled visits to any health care facility?…………□ times  **(Nanga maulendo ongopita opanda kuwuzidwa kuchipatala?**) | | | | | | | | | | | | |  |
| 3. How much does your supporter spend on scheduled visits for assessment for your most recent illness/follow up on:**(Kodi amene amakuprekezaniwo anagwilitsa ntchito ndalama zingati pa maulendo omwe adokotala adakuwuzani kuti mupite mukapimidwe m’mene munadwala chaposachedwapa?)**  **a)** Transport:___________________ **b)** Food:____________________  **c)** Accommodation:______________ **d)** Total Costs:____________________ | | | | | | | | | | | | |  |
| 4. How much does your supporter spend on unscheduled visits to any health care facility on:  **(Nanga adagwiritsa ntchito ndalama zingati pamaulendo ongopita kukalandira chithandizo kuchipatala?)**  **a)** Transport:………………………………… **b)** Food:…………………………………  **c)** Accommodation:……………………… **d)** Total Costs:………………………  **5. a)** Does your friend/family supporter have an income? Yes □ No □  **b)** If yes, how much per day?…………………………………………………………… | | | | | | | | | | | | |  |
| **6. a)** What was the main reason someone accompanied you?  **(Kodi chifukwa chenicheni ndichani chimene munthu wina anakuprekezani?)**  □Administrative barriers……… □Distance □Security □Too ill to travel alone  □Was required for treatment □Disability  □Other(Specify)___________________________  **b)** If Other, specify why_____________________________________________________ | | | | | | | | | | | | |  |
| ***H. HOSPITALISATION SECTION*** | | | | | | | | | | | | |  |
| 1. a) Have you been hospitalized for your most recent illness? **(Kodi munagonekedwa (admit) kuchipatala m’mene munadwala chaposachedwapa?)**  Yes □ No □  **If No, go to question I1**  **b)** How many days in total did you stay at the hospital? **(kodi munagona masiku angati pamodzi kuchipatala?)**□□□Days  C) How much did you pay in the hospital during your entire stay? (If nothing was spent, enter 0)  **(Kodi munapeleka ndalama zingati zonse pamodzi nthawi imene munali kuchipatala?)**  **i)** Total Cost:………………………………**ii)** Hospital Administration(consultation/Card Fee Cost:…………………………  **iii)**Bed Cost:………………………**iv)** Food Cost:……………………………………………………………………………  **v)** Transport Cost: …………………………… **vi)** Drugs:Cost:…………………………………………………………………………  **vii)** Other Cost:…………………………………  **d)** If Other Cost, specify what:……………………………………………………………………………………………………… | | | | | | | | | | | | |  |
| **2. a)** Did any family/friend stay with you while in hospital?**(kodi wachibale/nzanu anakhala nanu kuchipatalako?)** | | | | | | | | Yes □ No □**If No, go to question H3** | | | | |  |
| **b)** How many days in total did family/friend stay with you (sleep there)? □□□Days  **(Anakhala nanu masiku angati kuchipatala munthu ameneyu?)**  **c)** How much did your relative/friend pay for staying in the hospital? (If nothing was spent, enter 0)  **(Nanga munthu ameneyu agwiritsa ntchito ndalama zingati kuti akhale nanu kuchipatala?)**  **i)** Total Cost: ……………………………………… **ii)** Accommodation Cost: ……………………………………………………… **iii)** Food:Cost:……………………………………**iv)** Transport Cost:……………………………………………………………  **v)** Other Cost: ……………………………………  **d)** If Other Cost, specify what: ___________________________________________________________  **e)** Does your friend/family have an income?……Yes □ No □  **(munthu amene amakuthandiziraniyo kapena kupita nanu kuchipatala ali ndi njira yopezera ndalama?)**  **f)** If Yes, how much per day?___________________ | | | | | | | | | | | | |  |
| **3. a)** Did any other family/friend visit you  whileyou were in hospital?  ?**(Kodi wachibale aliyense kapena nzanu anabwela kudzakuwonani mutagonekedwa kuchipatala?)** | | | | | Yes □ No □**If No, go to question I1** | | | | | | | |  |
| **b)** If Yes, how many people visited you? □□Persons If forgotten by respondent, probe  **(Kodi ndi anthu angati omwe anabwera kudzakuwonani?)**  **c)** How many times did they visit you? □□Times If forgotten by respondent, probe  **(Odzakuwonaniwo anabwera maulendo angati?)**  **d)** What were the costs for your relative/friend who visited you in the hospital most recently?  (If nothing was spent, enter 0)  **(Kodi m’bale/mzanu yo anagwiritsa tchito ndalama zingati kuti abwere adzakuwoneni kuchipatala m’mene anabwera kudzakuwonani posachedwapa?)**  **i)** Total Cost: _______________________ **ii)** AccommodationCost: ___________________________  **iii)** Food:Cost:______________________ **iv)** Transport: Cost: ______________________________  **v)** Other Cost:_______________________  **e)** If Other Cost, specify what:_______________________________________________________  **f)** How long were the visits including travelling time? ……………hours……………minutes  **(Kodi maulendo amenewa amatenga nthawi yayitali bwanji kuphatikizapo nthawi yoyenda?)** …………… | | | | | | | | | | | | |  |
| ***K. PRODUCTIVITY LOSS*** | | | | | | | | | | | | |  |
| 1. a) Have you ever stopped working/going to school/doing housework due to your illness Symptoms? Yes No    **(Kodi munasiyako kugwira tchito/kupita kusukulu /kugwira ntchito zapakhomo chifukwa chamatendawa?**  b)If Yes, for how long? **(Mudasiya kwa nthawi yayitali bwanji?)**  1) Less than a month 2) 1 month 3) 2-3 months 4) 4-5 months 5) More than 6 months   1. **a)**Does someone stay home specifically to take care of you? Yes  No    **(Kodi alipo amene amangokhala kunyumba ndi cholinga choti adzikusamalirani basi?)**  **b)** If yes, for how long? _____________Weeks**(Kwa nthawi yayitali bwanji?)**  **c)** Did they quit their income-earning job to stay home and care for you? Yes No   **(Kodi anasiya tchito yawo imene imawabweretsera ndalama ndi cholinga choti adzikuyang’anirani?)**   1. How regularly did you work before you developed your lung symptoms?   **(Kodi ntchito munkagwira mochuluka bwanji musanapezeke ndi matendawa?)**  a) Throughout the year b) Seasonal/part of the year c) Day labour  d) Other (Specify)_________________________________________________   1. **a)** Did you have to change jobs when you developed this disease? Yes  No    **(Kodi munasitha ntchito mutapezeka ndizizindikiro za matenda amenewa?)**  **b)** What is your main occupation?:**(kodi mumagwira tchito yanji?):**  1) Farming 2) Business 3) Casual work 4) Employed 5) Student  6) Other (Specify):_______________________________________________________   1. **a)** How many hours did you work/study on average per week BEFORE you developed the lung symptoms?**(Kodi munkagwira tchito maola angati mwachidule pa sabata musanapezeke ndizizindikilo zamatendawa?)**   _________Hours per week  **b)** How many hours do you work/study on average NOW per week?**(Kodi mumagwira ntchito maola angati kapena kuphunzira maola angati mwachidule panopa pa sabata?)**  _________Hours per week   1. **a)** *If answer to 44 a) differs from question 44 b):* Is the change related to the lung symptoms?:   **(Kodi kusintha kwa maolaku kwachitika kamba ka kubwera kwa matendawa?)**  Yes  No   *b) If answer to 44 a) differs from answer to 44 b):* Is someone doing the work that you used to do? **(Kodi alipo amene akugwira tchito yomwe inuyo munkagwira?)**  Yes No   **c)** If Yes:**(Tsopano ndi ndani amene akugwira ntchito mmalo mwanu?)** 1) Daughter 2) Son 3)Spouse 4) Friend 5) Nobody 6) Other family | | | | | | | | | | | | |  |
